# 胸腺恶性肿瘤Masaoka-Koga分期相关术语的说明与定义

**DOI:** 10.3779/j.issn.1009-3419.2014.02.03

**Published:** 2014-02-20

**Authors:** Frank C. Detterbeck, Andrew G. Nicholson, Kazuya Kondo, Paul Van Schil, Cesar Moran, 北京大学肿瘤医院 付浩

**Affiliations:** 1 Division of Thoracic Surgery, Department of Surgery, Yale University School of Medicine, New Haven, Connecticut; 2 Department of Histopathology, National Heart and Lung Institute Division of Imperial College School of Medicine, London, United Kingdom; 3 Department of Oncological Medical Services, Institute of Health Biosciences, The University of Tokushima Graduate School, Tokushima, Japan; 4 Department of Thoracic and Vascular Surgery, Antwerp University Hospital, Antwerp, Belgium; 5 Department of Pathology, University of Texas MD Anderson Cancer Center, Houston, Texas

目前，胸腺恶性肿瘤尚无国际抗癌联盟（International Union Against Cancer, UICC）和美国癌症联合委员会（American Joint Committeeon Cancer, AJCC）的官方分期。在2017年新版国际肿瘤分期提出较为广泛接受的分期之前，国际胸腺肿瘤协作组织（International Thymic Malignancy Interest Group, ITMIG）仍然建议选用经Koga等修订的Masaoka分期^[1-3]^。然而，Masaoka和Koga分期都存在一些模糊的术语定义，尤其是对某些细节未作出明确的定义，造成学术界的许多混乱。为此，ITMIG首先由核心工作组起草推荐定义，再交由扩展工作组提炼，并于2010年11月16日ITMIG举办的定义和术语研讨会上进行了进一步修订，最终经ITMIG全体成员讨论后于2011年2月经ITMIG批准并被采用。其灵魂内容是ITMIG对Masaoka-Koga分期系统的许多细节问题给出了较明确定义与解释，旨在使得大家在应用Masaoka-Koga分期的过程中更加一致，以利于相互合作、资源共享，同时便于前瞻性数据的正确收集，最终提出更合理的分期系统供临床使用。本文就此作一综述。

## 1 分期系统的历史概述

1978年Bergh等^[4]^提出了一个分为三期的分期系统，Ⅰ期，肿瘤位于包膜内；Ⅱ期，肿瘤侵犯纵隔脂肪；Ⅲ期，肿瘤侵及周围器官或胸廓内转移。1年以后Wilkins等^[5]^提出了相似的分期系统，不同之处在于Ⅱ期中包括了纵隔胸膜或心包的侵犯。1981年Masaoka等^[1]^在对93例患者分析后提出了四期的Masaoka分期系统。1994年Koga等^[2]^对79例患者分析后对Masaoka分期进行了修订，修订后的分期系统得到了广泛应用。实际上对声称应用Masaoka分期的许多机构和作者再核查时，发现实际上应用的是Koga修订后的分期。此外，也有其他一些的分期系统，如修订Masaoka分期，将无镜下包膜侵犯的Ⅰ期根据有无粘连分为Ia和Ib两期^[6, 7]^。再如美国外科病理解剖学主任协会提出的根据是否侵犯大血管将Ⅲ期再进行细分^[8]^。一些作者推荐，Ⅲ期和Ⅳ期应考虑肿瘤是否完整的切除^[6, 7]^。1991年，一个法国的分期系统将是否完整切除纳入到胸腺恶性肿瘤的分期^[9]^，其实这更像是一个预后分期系统，因为作者除了肿瘤分期常用的焦点（解剖分期）外，还涉及了治疗结果。1991年Yamakawa等^[10]^分析了207例患者后提出了TNM分期，其中T分期遵循Masaoka分期，将孤立的胸膜或心包结节分为T4，Ⅳa期，而将任何有淋巴结或远处转移者归于Ⅳb期。此后，Tsuchiya等^[11]^于1994年和WHO于2004年对该分期进行了修订^[12]^。2005年Bedini等^[13]^对127例患者分析后提出了改良的TNM分期，即所谓的Istituto Nazionale Tumori分期，包括三期，Ⅰ期为局限性疾病，包括了Masaoka分期中的Ⅰ期和除纵隔胸膜侵犯外的Ⅱ期；Ⅱ期为局部进展性疾病，包括了侵犯局部结构或胸内淋巴结转移；Ⅲ期为全身性疾病，包括颈部淋巴结或胸外远处转移。然而，没有任何一种TNM分期被广泛接受，仅有针对Yamakawa分期系统的验证研究^[14, 15]^。

## 2 Masaoka-Koga分期系统

Masaoka-Koga分期系统见表 1。该分期的重点是对原发肿瘤局部侵犯的描述，而对淋巴结转移并没有放到重要位置，这可能是源于胸腺瘤淋巴结转移相对少见的缘故。但胸腺癌并非如此。

**1:** Masaoka-Koga分期系统 Masaoka-Koga staging system

分期	定义
Ⅰ	肉眼或镜下肿瘤包膜完整
Ⅱa	镜下侵透包膜
Ⅱb	肉眼侵犯正常胸腺或周围脂肪组织，或肉眼粘连但未侵透纵隔胸膜或心包
Ⅲ	肉眼侵犯邻近器官（如，心包，大血管，或肺）
Ⅳa	胸膜或心包转移
Ⅳb	淋巴或血行转移
摘自Pathol Int 1994; 44:359-367. 注：本表得到版权所有者© 2011 by the International Association for the Study of Lung Cancer复制许可。	

（1）Masaoka分期与Koga改良分期有以下几点区别：

● Koga分期将Ⅱ期定义为侵透包膜，而Masaoka分期对包膜侵犯描述的比较模糊。

●镜下侵犯周围脂肪组织在Koga分期中属于Ⅱa期，而在Masaoka分期中属于Ⅱb期。

● Koga分期明确提出了“与纵隔胸膜或心包粘连但未侵透”。

（2）Masaoka-Koga分期中的模糊定义：

●侵透包膜的确切含义是什么？

●缺乏完整包膜的肿瘤应该如何定义？

●肉眼侵犯但是镜下未侵犯（无论是胸腺周围脂肪还是邻近器官）的肿瘤应该如何分期？

●肉眼纵隔胸膜或心包粘连与侵犯有何区别？

●何谓与纵隔胸膜或心包粘连？

●如何区分侵犯和侵透纵隔胸膜？

●累及心包的程度在Ⅱb期和Ⅲ期中描述的不够清楚。

●怎样才能够知道孤立的肿瘤病灶何时已经发生了血行转移？

●该分期系统是否同样适用于胸腺癌？

Masaoka和Masaoka-Koga分期的主要争议是Ⅰ期和Ⅱ期间的生存差异小，而Ⅲ期涵盖的范围又太广，包括了从肉眼及镜下穿透胸膜的粘连到侵犯主动脉、肺动脉以及心脏的所有情况。本文的目的并不是定义一个新的、更好的分期系统，这需要前瞻性的研究以及细致的分析。目的是坚持既有的分期系统，但对细节进行定义，采用统一的方式记录前瞻性的数据，以便于更好评估这些数据。尽管目前采用的方法符合现行的分期标准，但对模糊概念进行明确定义对我们是很有帮助的。

Ⅰ期  胸腺瘤定义为未侵透包膜。侵及但未侵透包膜分为Ⅰ期，局限性胸腺瘤（表 2，图 1）。肿瘤必须侵透包膜才能定义为侵袭性胸腺瘤（此时不再为Ⅰ期）。诊断的具体操作过程在另一篇文章中讨论^[16]^。Ⅰ期胸腺瘤不能归为良性，就像原位癌不能被认为是良性一样。同时所有大宗病例长期随访都证实各种组织学类型的Ⅰ期胸腺瘤都可能出现复发和转移^[17]^。由于所有胸腺瘤都表现出这些恶性特征，因此所有胸腺瘤都被认为是恶性的（尽管大多数都是低级别的并可以获得成功的治疗）。在一些患者中，肿瘤包膜部分缺失，此时不能定为侵犯，应在报告中详细记录，例如，胸腺瘤伴部分包膜缺失，注明因包膜缺失包膜浸润不能被评估的区域，除非有肿瘤侵犯纵隔脂肪的明确证据，否则该肿瘤仍应被归为Ⅰ期。必须认识到包膜并不是肿瘤固有的解剖学标志，而是肿瘤引起的反应性纤维粘连，因此，区域性包膜缺失是可以存在的。

**2:** ITMIG对Masaoka-Koga分期的详细定义 ITMIG definition of details of the Masaoka-Koga Staging System

分期	定义（斜体字为ITMIG对细节的解释）
Ⅰ	肉眼和镜下肿瘤包膜完整
	*包括肿瘤侵犯但未侵透包膜、或* *肿瘤的包膜缺如但未侵犯周围组织*
Ⅱa	镜下侵透包膜
	*镜下侵透包膜（而非肉眼观察)*
Ⅱb	肉眼侵犯正常胸腺或周围脂肪组织，或肉眼粘连但未侵透纵隔胸膜或心包
	*肉眼肿瘤侵犯正常胸腺或胸腺周围脂肋（镜下怔实）或* *与胸膜或心包粘连，需一同切除，而且镜下证实有胸腺周围侵犯（但是镜下未侵犯或侵透纵隔胸膜或侵犯心包的纤维层)*
Ⅲ	肉眼侵犯邻近器官（如，心包，大血管，或肺）
	包括侵犯以下任何组织： *镜下侵犯纵隔胸膜（无论部分或侵透弹力蛋白层）；或* *镜下侵犯心包（无论部分侵犯纤维层或侵透浆膜层）或* *镜下证实直接侵透脏层胸膜或侵犯肺实质；或* *侵犯膈神经或迷走神经（镜下证实，仅粘连除外）；或* *侵犯或侵透大血管结构（镜下证实）；与* *肺或周围器官纤维粘连，且必须侵犯纵隔胸膜或心包（镜下证实)*
Ⅳa	胸膜或心包转移
	*镜下证实与原发肿瘤分开的肿瘤结节，位于脏层或壁层胸膜表明，或心包表面*
Ⅳb	淋巴或血行转移
	*任何淋巴结转移（如，前纵隔，胸腔内, 下颈部或前部淋巴结，任何胸外淋巴结)* *远处转移（如，胸外和颈部胸腺组织以外的淋巴结）或肺实质结节（而非胸膜种植)*
ITMIG：国际胸腺瘤合作组织。 注：本表得到版权所有者© 2011 by the International Association for the Study of Lung Cancer复制许可。	

**1:**
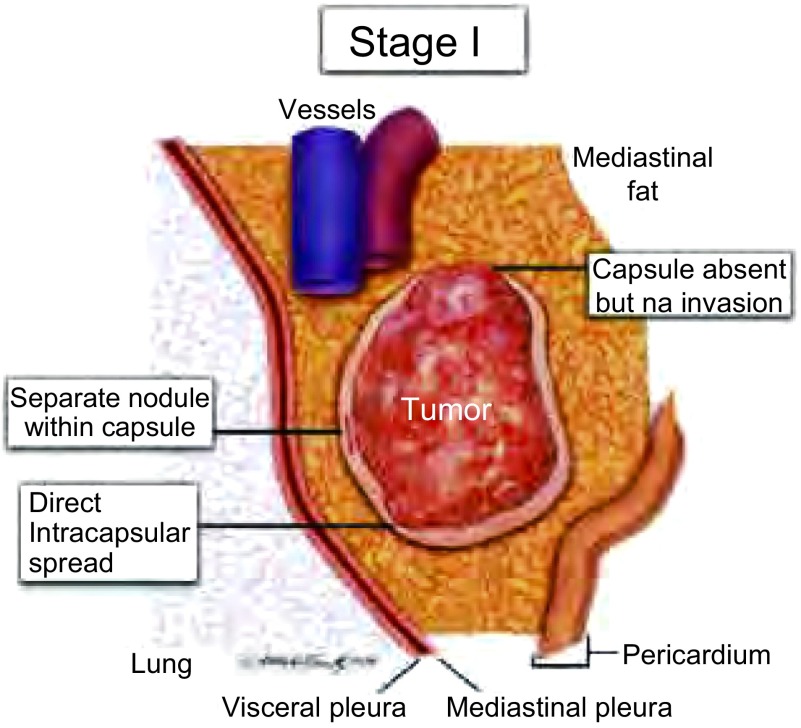
胸腺瘤侵犯或部分侵犯纤维包膜归为非侵袭性，包膜缺失不能归为侵袭性。 Penetrations within the fibrous capsule of a thymoma are classified as noninvasive, although they do (partially) invade the capsule. Absence of a capsule by itself does not constitute invasion.

Ⅱ期  侵透包膜的胸腺瘤应分为Ⅱ期。如果对包膜周围组织的镜下侵犯是局限的（例如，≤3 mm），那么肿瘤应该是Ⅱa期，微小浸润（图 2）^[16]^。如果证实肿瘤的浸润超出了包膜，不管侵及胸腺周围脂肪还是侵及胸腺瘤周围的正常胸腺，都应归为Ⅱ期。与之相反，如果肿瘤在包膜缺失的区域与邻近组织有简单的接触，那么肿瘤就不应该被定义为侵袭性，而应该被归为Ⅰ期。

**2:**
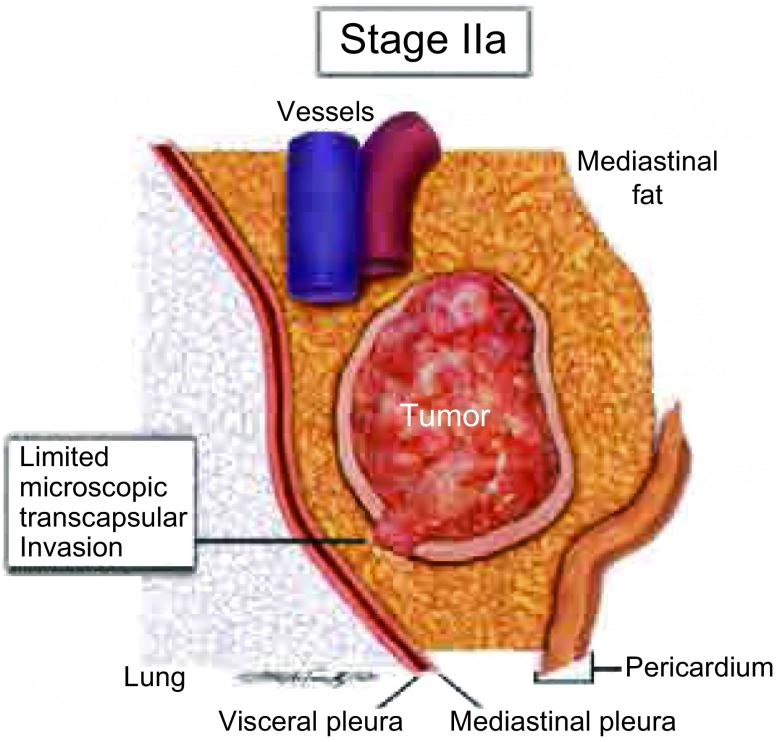
如图所示为侵透包膜的Ⅱa期胸腺瘤 Schematic diagram of transcapsular invasion included in stage Ⅱa

只有镜下证实对邻近结构有浸润才能被诊断为侵袭性胸腺瘤，如果只是肉眼怀疑浸润而没有得到镜下证实，那么最初的怀疑在最终分期时不予考虑。在收集前瞻性数据时必须注意到这一点，以便更好的完成对该分期的细化。最初的Masaoka分期系统对可疑侵犯不需要进行镜下确认^[1, 18]^。但是，ITMIG共识与其它恶性肿瘤的分期方法一致，其重点在于镜下诊断而非肉眼表现。此外，该共识对Masaoka-Koga分期系统作出了更加合理的修改，将Ⅱa期命名为镜下证实的微小浸润，Ⅱb期为镜下证实的肉眼侵犯。

Masaoka-Koga分期中Ⅱb期肿瘤是指镜下证实的肉眼侵犯到周围正常胸腺或纵隔脂肪组织（图 3A，图 3B）。建议还应该将侵及而没有侵透纵隔胸膜或心包的肿瘤包括在内，但这可能难以区分。不论何时，如果肿瘤延伸到临近纵隔胸膜或心包而没有浸润，那么应该注明肿瘤与纵隔胸膜或心包的距离。评估的具体操作流程在另一篇文章中进行讨论^[16]^。

**3:**
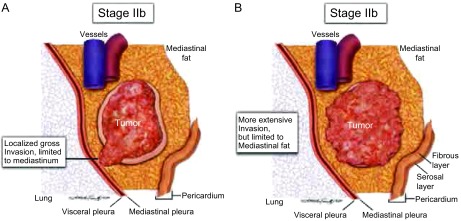
A、B所示为Ⅱb期胸腺瘤。A图可见局部区域侵犯；B图可见广泛累及周围纵隔脂肪组织，但未累及胸膜或心包。 A and B, Types of invasion included in stage IIb. This may range from (A) a single area of localized invasion to (B) more extensive involvement of the mediastinal fat without pleural or pericardial involvement.

根据有无纵隔胸膜或心包侵犯来鉴别Ⅱb期和Ⅲ期，因为这与现有的Masaoka-Koga分期是一致的（Ⅲ期：肉眼侵犯…心包）。此外，定义部分侵犯或侵透纵隔胸膜或心包是很困难的。最后，过去鉴别纵隔胸膜的标准是不一致的，任何试图对这一结构的一致性给予重视的尝试都代表着某种进步^[16]^。解决该问题需要前瞻性研究，使得分期客观并有数据支持。

如果肉眼观察怀疑肿瘤侵犯纵隔胸膜或心包，但未得到镜下证实，肿瘤应该被注明为粘连，但是要根据镜下表现决定是分为Ⅰ期、Ⅱa期还是Ⅱb期。在病理报告中应该被描述为“肿瘤粘连至但未侵犯”纵隔胸膜或心包。由于对粘连的精确定义难以表达清楚，建议粘连应该是肿瘤在肉眼观察时与纵隔胸膜或心包紧密接触并且需要切除。区分不伴有侵犯的单纯粘连和镜下侵犯纵隔胸膜或心包，并不是Masaoka或Koga分期系统的初衷^[1, 18]^。但为了与其他恶性肿瘤分期相一致，需要做出这种解释，这代表了ITMIG会员的共识。关于这一问题，也需要前瞻性的评估。

Ⅲ期  Masaoka-Koga分期中的Ⅱb期和Ⅲ期对心包的描述比较模糊。我们建议所有侵及（不管是部分侵犯还是侵透）纵隔胸膜或心包的肿瘤都应该被分为Ⅲ期，前瞻性的数据在收集的时候都应该注意这些细微差别。如果仅仅是侵犯到心包的纤维层或侵透浆膜层或心包浆膜层的表面，那么在病理报告中必须予以注明。纵隔胸膜是一种含实质较少的结构，建议邻近肿瘤的弹力层不连续定为纵隔胸膜受侵犯的证据（图 4A，图 4B）。

**4:**
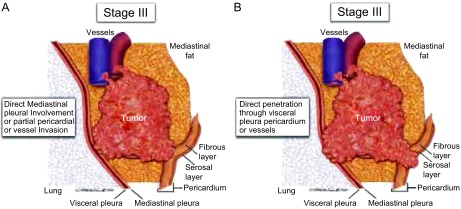
A、B所示为Ⅲ期胸腺瘤。A图可见肿瘤侵犯纵隔胸膜，或部分侵犯心包或血管；B图可见肿瘤侵透心包、脏层胸膜或膈神经。 A and B, Types of invasion included in stage Ⅲ. A, Schematic diagram of involvement of the mediastinal pleura or partial involvement of the pericardium, or vessels.B, Penetration through the pericardium, the visceral pleura, or into the phrenic nerve.

肿瘤穿透胸膜腔侵犯到脏层胸膜也应该归为Ⅲ期。如果脏层胸膜被破坏，应该在病理报告中注明，必要时可应用弹力蛋白染色。肿瘤侵犯到肺实质或侵犯无名静脉或其它血管结构，不管是部分侵犯还是侵透，只要镜下证实就应归为Ⅲ期。尽管肿瘤侵犯膈神经或迷走神经在Masaoka-Koga分期中没有详细说明，建议将其归为Ⅲ期。受侵犯的器官以及侵犯的程度（侵及或侵透）应该被记载以便进一步研究。

Ⅲ期应遵循的原则是镜下证实，而不能仅依靠肉眼诊断。因此，仅仅是与肺、膈神经或心包等结构粘连而缺乏镜下证实的侵犯不能分为Ⅲ期。只有侵犯到心包内结构时，才能肉眼诊断Ⅲ期。主动脉、上腔静脉或肺动脉外表面的孤立种植结节，应归为Ⅳa期。

Ⅳa期  孤立于原发肿瘤的胸膜或心包肿瘤结节应归为Ⅳa期。这些孤立的肿瘤结节可以位于脏层或壁层胸膜或心包或心外膜表面（图 5A）。胸腺恶性肿瘤直接侵犯到心包或胸膜表面，而非孤立的结节应归为Ⅲ期。

**5:**
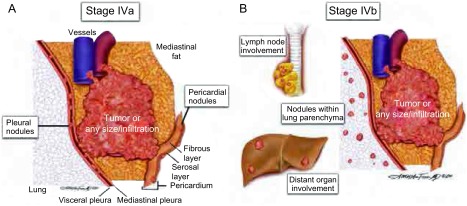
A、B所示为Ⅳ期胸腺瘤。A图可见胸膜或心包表面的孤立性结节，归为Ⅳa期；B图可见淋巴结转移、胸腔外转移或肺内结节。 A and B, Separate foci of tumor included in stage Ⅳ. A, Separate nodule on the pleural or pericardial surfaces classified as Ⅳa. B, Involvement of nodal sites, extrathoracic sites, or parenchymal lung nodules.

Ⅳb期  肿瘤累及胸腺周围淋巴结为Ⅳb期。这包括前纵隔、气管旁和隆突下淋巴结以及沿胸腺上极走行的淋巴结。纵隔其它部位的淋巴结或胸腔内的淋巴结转移也应归为Ⅳb期。胸腔外淋巴结（邻近胸腺的颈部淋巴结除外）应该考虑为远处转移（但是并不会改变分期），这与其它肿瘤的定义相一致（例如肺癌）。

结节位于肺组织内，且结节和胸膜表面之间有正常肺组织被定义是远处转移。累及胸腔外组织（邻近胸腺的颈部区域除外）也应归为远处转移（图 5B）。

推荐应用的术语是肺转移和胸腔外转移而不是血行转移。前者描述的是实际的解剖学位置而后者指的是纯理论上的扩散机制。没有数据来证实所推测的机制，越来越多来源于其它肿瘤的人体数据表明转移扩散机制是复杂的，它涉及到粘附、迁移、种植、血管生成等，不单纯是血液中出现肿瘤细胞。

ITMIG虽然对Masaoka-Koga分期作了详细的解释，但需要前瞻性研究来证实。这就需要一个足够详细数据库来研究这些细节问题。需要统一的标准收集数据，从起点上达成一致。这里所推荐的定义就代表了这样一种统一，这些定义可能存在武断性且很大程度上尚未得到证实，但是将来一旦获得充分的数据则会发生实质的改变。推荐的定义主要集中在病理学分期上，因为这也是Masaoka-Koga分期的焦点所在。我们认识到制定更好的术前临床分期的必要性，但是本文只讨论对一种既存分期系统作出一致性解释的必要性。临床分期的意义需单独讨论。

ITMIG所采用的定义与之前的Masaoka-Koga分期有一些小的差别。之前发表的文章没有明确定义纵隔胸膜或心包侵犯的范围，对界线的划分存在争议。事实上，在ITMIG讨论之初也是存在这些争议的，但最后所有成员统一提出了该争议的明确定义。也许，与之前的Masaoka和Koga分期最明显的区别在于本文提出的分期规定肉眼怀疑的侵犯必须得到镜下证实。实际上其它肿瘤分期都是采用的这种标准，因此不推荐胸腺瘤在该问题上采用其它分期标准。

ITMIG推荐将Masaoka-Koga分期应用于胸腺瘤和胸腺癌（包括胸腺类癌瘤和其他胸腺肿瘤的少见类型）。当病例数据缺乏时，最好的方法是保持数据的一致性和简单化。因为胸腺癌的淋巴结转移较胸腺瘤更为常见，这种分期方法也许是值得怀疑的，但提出两种不同分期方法应该基于强有力的研究数据。
